# Harnessing the Utility of *Ex Vivo* Patient Prostate Tissue Slice Cultures

**DOI:** 10.3389/fonc.2022.864723

**Published:** 2022-03-31

**Authors:** Lillian M. Perez, Larisa Nonn

**Affiliations:** ^1^University of Illinois at Chicago Pathology Department, Chicago, IL, United States; ^2^University of Illinois Cancer Center, Chicago, IL, United States

**Keywords:** prostate, *ex vivo* culture, precision medicine, prostate cancer, androgens

## Abstract

Patient-derived prostate tissue explant cultures are powerful research tools that offer the potential for personalized medicine. These cultures preserve the local microenvironment of the surrounding stroma but are not without limitations and challenges. There are several methods and processing techniques to culture tissue *ex vivo*, that include explant tissue chunks and precision-cut tissue slices. Precision-cut tissue slices provide a consistent distribution of nutrients and gases to the explant. Herein we summarize the prostate tissue slice method, its limitations and discuss the utility of this model, to investigate prostate biology and therapeutic treatment responses.

## Introduction

*Ex vivo* tissue culture retains the local microenvironment and is potentially a powerful tool to examine prostate responses to treatment and/or genetic manipulation. This technique is particularly relevant to prostate, which contains several cell types including glandular epithelium, fibromuscular stroma, neuroendocrine cells, and immune cells. The crosstalk between these cell types may influence experimental responses between patients. Several methods for *ex vivo* prostate tissue culture have been reported, which have some similarities, but there is no established gold standard method. Briefly, prostate tissue, benign or cancer, is cultured in media within a culture vessel over the course of 2-5 days. The patient-derived explant (PDE) model typically refers to prostate tissue that is chopped, minced, or sliced with surgical tools whereas tissue slice culture (TS) utilizes a precision slicing method to cut slices into an exact thickness. These methods are also useful for *ex vivo* patient-derived xenografts (PDXs) from mice. This review focuses on the prostate TS method, utilization, challenges and opportunities.

## *Ex vivo* Radical Prostatectomy Tissues Cultured as Precision Cut Slices

Precision cut slices from fresh tissues enable consistent diffusion of gases across the tissue and rely on capillary action to bring culture medium into the tissues. Slices were initially developed using liver and kidney for use in pharmacology metabolism studies (1). Parrish et al. were the first to extend this method to other tissues, including prostate (2). The *ex vivo* culture of prostate TSs was further refined to preserve the secretory epithelium and reduce basal cell hyperplasia (3). Androgen responsiveness is essential for any *ex vivo* model and the Peehl Lab showed that TS respond to androgens and androgen ablation both *ex vivo* and when grafted under the renal capsule of mice (3, 4).

TS relies on precise sectioning of a core of fresh radical prostatectomy tissue using a specialized tissue slicing instrument. The uniform thickness of the slices enables even nutrient and oxygen diffusion through the tissue to avoid necrosis (3). Diseases of the prostate, such as cancer, are often multi-focal and challenging to identify on gross specimens, thus it is essential to collect slices for histological examination. Culture length is variable and has been reported between one day and five days, dependent on endpoints.

## Tissue Slice Culture Method

**Figure 1:**
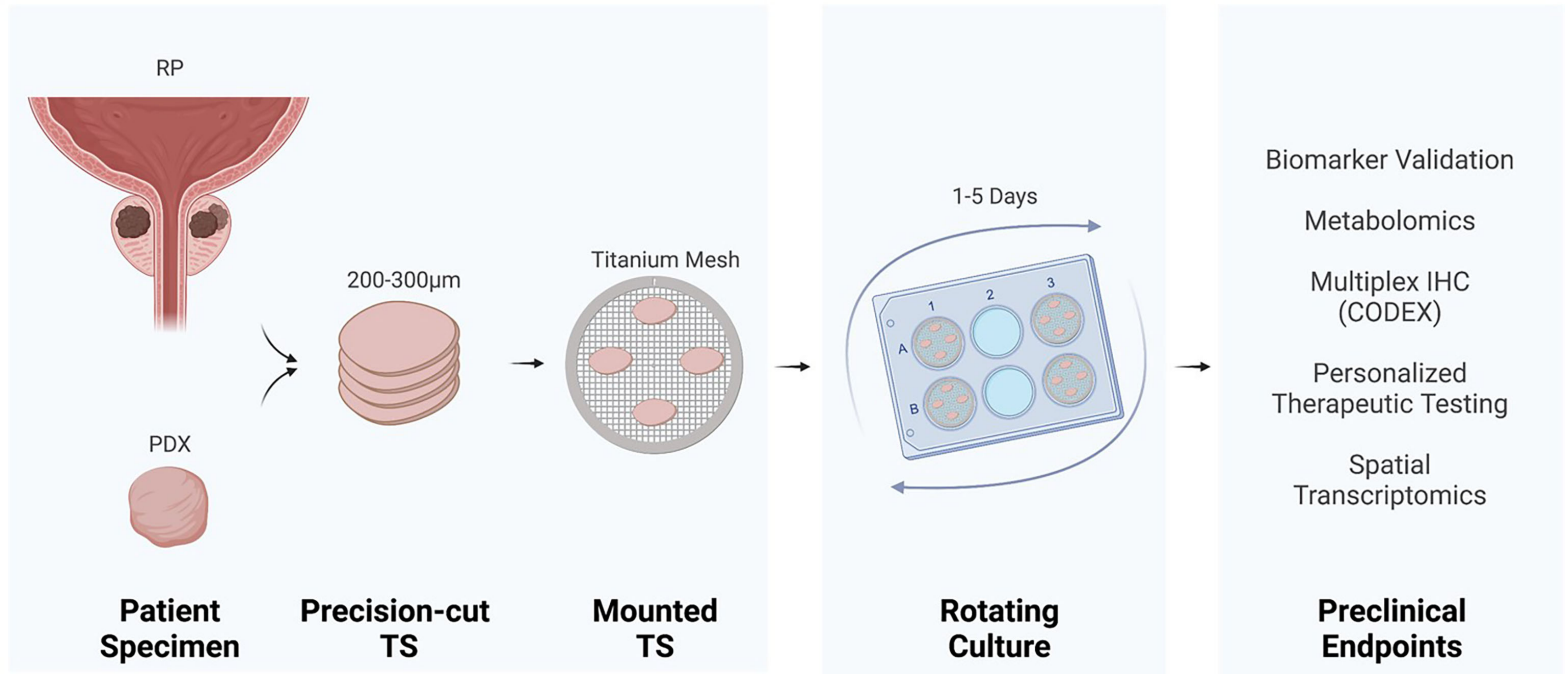
Tissue slice culture workflow and preclinical endpoints. Precision cut tissue slices (TS) derived from radical prostatectomy (RP) or a patient-derived xenograft (PDX) incubate on titanium mesh grids at a 45° angle within a rotating tissue culture plate.

Patient radical prostatectomy specimens or PDXs (5) have been used for prostate TS cultures (**Figure 1**). The detailed method has been reported by others (2, 4, 6, 7). Briefly, a 5 or 10 mm core of fresh tissue is stabilized in agar and mounted in a precision slicer, generating ~300 µm slices, which are quickly placed into culture. The majority of studies utilize titanium mesh inserts to mount slices within 6-well tissue culture plates (3, 6, 8–12). The TS on a titanium mesh rotates on an angle to dip the TS in and out of media, driving capillary action for equivalent distribution of media and exposure to gases. However, earlier tissue cultures have utilized titanium mesh within scintillation vials (2). Alternative to mounting TS on titanium mesh, Blauer et al. demonstrated retention of androgen responsiveness and luminal epithelium by culturing the TS completely submerged (7).

Optimization of primary culture medium can be challenging and TS cultures have been tested in different mediums including KSFM, M199, MCD105, and PFMR, with additives of supplements, serum, and androgens. One of the earliest studies used KSFM on titanium mesh within scintillation vials and varied supplementation with bovine pituitary extract, EGF, DHT, and FBS (10%) (2). They concluded that DHT promotes tissue slice viability, as the medium containing DHT prevented the loss of luminal epithelial cells, and that media containing 10% FBS promotes hyper-proliferation of basal cells. This proliferation of basal cells was also observed by Maund et al., who found low levels of androgen resulted in the basal hyper-proliferation (3). Maund et al. systematically tested many conditions for TS cultures. The first condition contained a mixture of KSFM and M199 [the 1:1 ratio as reported by (7)] with 1 nM DHT and resulted in luminal cell degeneration and basal cell hyperplasia. The second media contained PFMR-4A with 10 nM R1881 and resulted in less cell loss and viability for up to two days, after-which tissue slices exhibited luminal cell degeneration and basal cell hyperplasia. The third media contained PFMR-4A with 50 nM R1881 and resulted in the most cell viability at two days, with histology, proliferation, and apoptosis that was most similar to day zero TS. In summary, assessment of viability, toxicity, proliferation, and apoptosis resulted with the media composition containing PFMR-4A and 50 nM R1881 being optimal for TS culture media and concentration of testosterone (3). However, PFMR-4A is not commercially available and others have found success using PrEGM supplemented with 50 nM R1881 (12) or serum‐free aDMEM/F12 K medium with R1881 (13).

TS from patient derived xenograft models (PDX-TS), has been used in several studies. Zhang et al. did a systematic analysis on PDX-TS culture method and the effects of media composition (13). They used 3 PDX-TS and found that rocking on a cell strainer (very similar to titanium grids rotating) in serum‐free aDMEM/F12 K medium with R1881 was optimal to preserve proliferation and prevent apoptosis. Proof of concept studies in PDX-TS have developed a method to examine many tissues at once in 96-well format by a method they call micro-dissected tissue (MDT) (14, 15). MDT are 500 µm in diameter, compared to normal TS which are 3-5 mm, thus MDT permits more precise tissue acquisition and potentially many more experimental endpoints. Dorrigiv et al. optimized this by using cell line xenografts as the tissue source (15). They also developed a method to create an FFPE microarray from the MDT (MDTMA) (14). The small MDT appeared to be free of the challenges with larger tissue pieces and preserved cell morphology, viability, and proliferation throughout 15 days of culture.

## Investigation of Prostate Biological Responses Using Tissue Slice Cultures

TS cultures have been utilized to target key developmental signaling pathways and validate prior findings from *in vitro* studies in human prostate. TS retain expression of the hormone receptors and are shown to be responsive to the hormones vitamin D (12, 16) and androgen (2, 3, 7, 9, 13, 16, 17). McCray et al. analyzed TSs treated with 25-hydroxyvitamin D and analyzed epithelial and stromal gene expression *via* spatial transcriptomics (12). Among a panel of genes, the Wnt pathway and DKK3 were identified as downregulated by 25-hydroxyvitamin D. In another study, TS responded to both 25-hydroxyvitamin D and testosterone in regulation of target genes (16). The TS also retained expression of the endocytic membrane receptor, megalin, which imports hormones into the cells (16). Importantly, in both vitamin D studies, the TS data corroborated evidence from patient-derived organoids and relationships in patient specimens, demonstrating consistency and reproducibility between models to investigate developmental pathways *ex vivo*.

Several studies have utilized TS cultures to investigate DNA damage responses. DNA damage response of benign radical prostatectomy-derived TS was monitored following ionizing radiation (IR) (6). They found that IR did not elicit Tyr (15) and p53 responses in TS and they suggest that absence of these DNA damage response pathways may contribute to carcinogenesis (6).

## TS and Biomarker Discovery

TS from prostate cancer (PCa) are suited for biomarker discovery since the amount of tissue of each pathology can be controlled; matched benign/PCa tissues are available from each patient, and specimens from multiple patients can be used to account for inter-patient heterogeneity. Spichiarich et al. identified glycoproteins, specifically sialylated glycans, associated with PCa using bio-orthogonal labeling (10). They used paired benign and PCa TS from eight different patients and identified 21 proteins unique to all PCa samples and undetected in the benign tissue, including VDAC1 and the sialoglycoprotein, legumain. The unique metabolic state of PCa compared to benign tissue is preserved in TS and a source for biomarkers. The Kurhanewicz group used intracellular labeling of [^3-13^C] pyruvate in TS (7 benign and 4 PCa) to identify hyperpolarized ^13^C lactate as a PCa biomarker (8). They further examined lactate by TS of various Gleason Grade and showed that high-grade PCa (N=4) has higher lactate than low grade (N=11) or benign tissue (N=15) (11).

## Therapeutic Responses In *Ex Vivo* PDE and TS

Research advancements in anti-androgen therapies, alternative therapeutics for CRPC models, and other experimental therapeutics have been made with *ex vivo* TS or PDEs (**Table 1****)**. PDE cultures of tissue chunks have been used more frequently than TS for these studies. Although there are limited reports that use TS to measure treatment outcomes, these reports have demonstrated responses that may inform treatments in the clinic. PDEs are described as the *ex vivo* culture of prostate tissue as small pieces on sponges with conditions that promote or maintain tumor microenvironment and tissue architecture. *Ex vivo* PCa tumor cultures have demonstrated the tumor microenvironment remains intact using established methods (21). Both low and high tumor grades (Gleason 3-5) have been cultured successfully and remain viable for up to five days (3, 8, 10, 11). The methodology for PDE cultures and the promise of their preclinical utility has been previously reviewed by others (20, 28, 29) and is only briefly discussed here.

**Table 1:** Therapeutics tested in *ex vivo* prostate cultures.

Target	Therapeutic	*Ex Vivo* Model	Reference
ACC1/2	PF-05175157 (10 µM, 25 µM, 50 µM)	PDE	Butler et al., 2021 (18)
AR	Apalutamide (1 µM) + EBRT (2Gy)	PDX (TS, 300 µm)	Zhang et al., 2019a (19)
	Bicalutamide (10 µM)	PDE	Centenera et al., 2013 (20)
	Castration	TS grafts, 300 µm	Zhao et al, 2013 (9)
	Enzalutamide (1 µM)	PDE	Shafi et al., 2018 (21)
	Enzalutamide (1 µM)	PDX (TS, 300 µm)	Zhang et al., 2019b (13)
	Enzalutamide (1 µM) + Docetaxel (50 nM)	PDE	Shafi et al., 2018 (21)
	Enzalutamide (10 µM, 50 µM)	PDE	Butler et al., 2021 (18)
	Enzalutamide (10 µM)	CRPC-PDX (PDE)	Lawrence et al., 2018 (22)
	Enzalutamide (10 µM)	PDE	Boibessot et al., 2021 (23)
	Enzalutamide (10 µM)	PDE	Centenera et al., 2013 (20)
	Enzalutamide (10 µM)	PDE	Centenera et al., 2021 (24)
	Galeterone (10 µM)	CRPC-PDX (PDE)	Lawrence et al., 2018 (22)
BCL-2	Cisplatin + ABT-737 (10 µM)	TS, 200 µm	Bray et al., 2009 (25)
BRET	iBET151 (1 µM) and JQ1 (1 µM)	CRPC-PDX (PDE)	Lawrence et al., 2018 (22)
CDK4 and CDK6	Palbociclib (1 µM)	PDE	Shafi et al., 2018 (21)
	Ribociclib (1 µM)	CRPC-PDX (PDE)	Lawrence et al., 2018 (22)
DNAPK	NU7441 (1 µM)	PDE	Shafi et al., 2018 (21)
HSP90	NVP-AUY922 (100-1000 nM)	PDE	Centenera et al., 2013 (20)
	NVP-HSP990 (100-1000 nM)	PDE	Centenera et al., 2013 (20)
pan-PIM	CX-6258 (5 µM)	CRPC-PDX (PDE)	Lawrence et al., 2018 (22)
PARP	Talazoparib (1 µM)	CRPC-PDX (PDE)	Lawrence et al., 2018 (22)
	ABT888 (2.5 µM)	PDE	Schiewer et al., 2012 (26)
	Olaparib (10 µM)	PDX (TS, 300 µm)	Zhang et al., 2019 (19)
	Veliparib (2.5 µM)	PDE	Shafi et al., 2018 (21)
RNA polymerase I	CX-5461 (1 µM)	CRPC-PDX (PDE)	Lawrence et al., 2018 (22)
STAT3	Galiellalactone (5 µM)	PDE	Handle et al., 2018 (27)

TS, tissue slice culture; EBRT, external beam radiation therapy; PDX, patient-derived xenograft; PDE, patientderived explant; CRPC, castration resistance prostate cancer.

TS and PDEs are highly responsive to androgens and have been used to examine several anti-androgen therapies. Zhao et al. revealed that the castration response of TS grafted into mice mimicked the expression of proteins in prostate specimens from patients with androgen deprivation therapies (9). The patient heterogeneity of anti-proliferation responses to bicalutamide and enzalutamide was demonstrated in PDE (17, 18). Butler et al. further showed tumor areas resistant to enzalutamide also had aberrations in their lipid profiles (18), which lead to discovery of ELOVL5, a fatty acid elongase, as a new metabolic target of androgens (24). The potential to predict patient response to combination therapy with enzalutamide and docetaxel was shown in PDE, reflecting individual treatment outcomes observed in the clinic (21). Shafi et al. also identified heterogeneous responses to experimental therapeutics, including veliparib, palbociclib, and NU7441 (21), emphasizing that patient-specific responses may be tested in PDEs. The antiandrogen therapy, apalutamide, was shown to radiosensitize PDX-TS PCa demonstrating a possible therapeutic treatment for AR-dependent PCa (19). Explants are also useful for studying resident immune cells and PDEs were recently used to show an increase in CD163+/CD68+ macrophages after enzalutamide (23).

PDEs from PDXs have been used to test therapeutics. Galiellalactone, a STAT3 inhibitor, reduced AR activity in PDEs of thin tissue pieces (cut by a razor blade) (27). Bray et al. showed in 5 human prostatectomy-derived tumors that combination treatment with BCL-2 inhibitor, ABT-737, and cisplatin yielded a synergistic therapeutic response more than either treatment alone using *ex vivo* PCa TS (200 µm) (25). PDEs from PDX of CRPC demonstrated sensitivity of CRPC to BRET inhibitors (22), and PARP inhibitors (26). Zhang at al. used PDXs from 3 patients for TS cultures and observed the expected responses to enzalutamide and olaparib, based on the AR expression and BRCA2 mutations, respectively (13).

## Limitations and Challenges of Prostate TS and *Ex Vivo* Cultures

There are challenges that are common between all *ex vivo* culture methods and those unique to TS. The primary challenge to *ex vivo* cultures for TS (or PDEs) is rapid accessibility to fresh surgical specimens. This requires close collaboration and cooperation between research and clinical staff as well as pre-surgical consent of the patient to utilize excess tissue not needed for diagnosis. The size of the specimen further limits the amount of TS or PDE that can be made from it. These challenges contribute to the rarity of *ex vivo* CRPC cultures and underscore the importance of PDX-CRPC. To date, neuroendocrine prostate cancer (NEPC) has yet to be reported for TS studies, likely owing to limited access. Identification of cancer areas on gross radical prostatectomy specimens is a challenge. Furthermore, fresh tissue pieces are never one cell type. Benign areas will contain varied amounts of glandular epithelium and stroma. PCa often presents multi-focal lesions that are not fully encapsulated in a sample. Thus, samples for *ex vivo* culture may contain mixed pathologies that will bias endpoints that homogenize the entire piece of tissue. The TS method preserves histological features and allows for spatial examination of endpoints such as immunohistochemistry, *in situ* hybridization, ISH or spatial transcriptomics. The method of MDT (described above) (14) and pathology guided micropunching (PGM) (30) sample smaller areas (260-500 µM), reducing heterogeneity of the specimens, but also provide limited tissue for endpoint analyses. The relatively short length of culture for androgen adaptation studies is a limitation to both PDEs and TS. However, several studies grafted TS under in the renal capsule of nude mice, rather than *in vitro* culture, and were able to predict androgen sensitivity (4, 9). Finally, while TS are optimal for assessing therapeutic responses, overexpression and knockdown tools needed for mechanistic studies need to be carefully optimized to penetrate into explant tissue cultures. This includes delivery of siRNAs, as reported in localized PCa PDE cultures by Tieu et al. (31).

TS have several additional limitations. The main one is that specialized equipment is required to prepare and culture the precision slices. Secondly, as PCa tumors are small, the pathology of the specimen often drifts through the slices, requiring additional collection of slices for pathology if the endpoint doesn’t facilitate visualization of the histology. Lastly, preparing frozen or FFPE sections from the TS is challenging and requires a trained technician to obtain high quality sections from a TS that is only about 200 µM thick after fixation.

## Opportunities for Prostate TS *Ex Vivo* Culture

Despite the challenges, TS cultures provide spatial examination of inter and intra-patient heterogeneity not possible by other methods. The rapid advancement and increased resolution of spatial transcriptomic methods (32, 33) offers the ability to compare transcriptomic differences (RNAseq) between areas of the tissue and between patients. Co-detection by indexing (CODEX) tissue imaging with DNA-barcoded antibodies has the potential to examine up to 60 markers in one sample (34), which would greatly expand the data available from a TS experiment. Localized prostate tumors are often under hypoxia and Figiel at al recently showed that localized PCa TS respond to hypoxic culture conditions (35), which support use of TS in therapeutic response studies.

In summary, although *ex vivo* culture of prostate TS was first described two decades ago, it remains an emerging model that holds promise for both research questions and for precision medicine. TS are primed and amenable to recent technologic breakthroughs in single cell sequencing and spatial data collection.

## Author Contributions

LMP and LN jointly wrote and edited this manuscript. All authors contributed to the article and approved the submitted version.

## Funding

This work was supported by Department of Defense Prostate Cancer Research Program Cancer Health Disparities Grant PC190699 (LN) and University of Illinois Cancer Center Cancer Biology Training Program Pilot Funding (LP).

## Conflict of Interest

The authors declare that the research was conducted in the absence of any commercial or financial relationships that could be construed as a potential conflict of interest.

## Publisher’s Note

All claims expressed in this article are solely those of the authors and do not necessarily represent those of their affiliated organizations, or those of the publisher, the editors and the reviewers. Any product that may be evaluated in this article, or claim that may be made by its manufacturer, is not guaranteed or endorsed by the publisher.
